# Serum levels of gamma-glutamyltransferase predict outcome in heart failure with preserved ejection fraction

**DOI:** 10.1038/s41598-019-55116-8

**Published:** 2019-12-06

**Authors:** Daniel Dalos, Christina Binder, Franz Duca, Stefan Aschauer, Andreas Kammerlander, Christian Hengstenberg, Julia Mascherbauer, Thomas Reiberger, Diana Bonderman

**Affiliations:** 1 Department of Internal Medicine II, Division of Cardiology, Medical University of Vienna, Vienna, Austria; 2 Department of Internal Medicine III, Division of Gastroenterology and Hepatology, Medical University of Vienna, Vienna, Austria; 3 Hepatic Hemodynamic Laboratory, Medical University of Vienna, Vienna, Austria

**Keywords:** Heart failure, Hepatology

## Abstract

Previous studies suggested an association between heart failure (HF) and hepatic disorders. Liver function parameters have been shown to predict outcome in HF with reduced ejection fraction, but their impact in HF with preserved ejection fraction (HFpEF) has not yet been investigated. Between January 2011 and February 2017, 274 patients with confirmed HFpEF were enrolled (age 71.3 ± 8.4 years, 69.3% female) in a prospective registry. During a median follow-up of 21.5 ± 18.6 months, 97 patients (35.4%) reached the combined endpoint defined as hospitalization due to HF and/ or death from any cause. By multivariable cox regression, serum gamma-glutamyltransferase (GT) was independently associated with outcome (Hazard Ratio (HR) 1.002, p = 0.004) along with N-terminal pro brain natriuretic peptide (HR 2.213, p = 0.001) and hemoglobin (HR 0.840, p = 0.006). Kaplan-Meier analysis showed that patients with serum gamma-GT levels above a median of 36 U/L had significantly more events as compared to the remainder of the group (log-rank p = 0.012). By multivariable logistic regression, higher early mitral inflow velocity/ mitral peak velocity of late filling (Odds Ratio (OR) 2.173, p = 0.024), higher right atrial (RA) pressure (OR 1.139, p < 0.001) and larger RA diameter (OR 1.070, p = 0.001) were independently associated with serum gamma-GT > 36 U/L. Serum levels of gamma-GT are associated with both left and right-sided cardiac alterations and may serve as a simple tool for risk prediction in HFpEF, especially when further diagnostic modalities are not available.

## Introduction

In patients presenting with heart failure (HF), left ventricular (LV) ejection fraction (EF) will be normal in 50% of cases. Together with elevated natriuretic peptides and structural changes, such as left atrial enlargement, the diagnosis of HF with preserved ejection fraction (HFpEF) can be made^1^. On a pathophysiological level, abnormal LV relaxation results in elevated filling pressures due to changes in cellular as well as collagen metabolism^2–5^. Affected patients suffer from dyspnea, exercise intolerance and impaired quality of life. In advanced stages, they also show signs and symptoms of central and/or peripheral congestion and face a dismal prognosis, similar to that of HF patients with reduced ejection fraction^6,7^.

Increasing evidence suggests an association between HF and hepatic disorders. In a meta-analysis published in 2000, Naschitz *et al*. classified this organ interaction according to etiology^8^. Heart diseases that are linked to liver alterations include congestive fibrosis or cardiogenic ischemic hepatitis. Liver disorders resulting in cardiac impairments are classified as cirrhotic and non-cirrhotic complications. Complications of cirrhosis include hepatopulmonary syndrome or pericardial effusion, whereas a non-cirrhotic complication may be high-output failure caused by intrahepatic arteriovenous fistulae. Combined disorders with common etiology may be caused by infectious, metabolic, immune or toxic conditions^8^.

In acutely decompensated HF (ADHF) patients with reduced EF and in patients with cardiogenic shock, abnormal liver function tests have previously been described and were independently associated with poor outcome^9–15^. For example, in the SURVIVE study observing ADHF patients elevated transaminases were found in 46% of patients - and were associated with a 2-fold increase in 31-day mortality^14^. In the EFICA trial studying patients with cardiogenic shock elevations in transaminases were an independent predictor of 4-week mortality^15^.

In addition, a prognostic value of serum bilirubin has been suggested in the acute^16,17^ and the chronic phase of HF^18,19^ as well as in patients with idiopathic pulmonary hypertension^20^.

Although there is some evidence that non-alcoholic fatty liver disease (NAFLD) is associated with LV diastolic dysfunction in patients with diabetes^21^, the prognostic role of liver enzymes in HFpEF as well as their pathophysiological correlates have not been investigated so far. Therefore, the aim of this study was to describe the association of commonly assessed liver enzymes with clinical outcome in patients with HFpEF. Furthermore, we sought to identify alterations of cardiac structure and function that may underlie deviations of liver function parameters.

## Methods

### Subjects and study design

This prospective, observational cohort-analysis was performed at the Division of Cardiology of the Medical University of Vienna, a tertiary referral center for HFpEF. Approval from the ethics committee from the Medical University of Vienna was obtained before study initiation (EK #796/2010). All procedures were performed in accordance with the relevant guidelines and regulations. Written informed consent was obtained from all patients prior to enrollment and any study-related procedure.

Consecutive patients with HFpEF were included from the outpatient department. The following examinations were performed: physical examination including bioelectrical impedance spectroscopy, 12-lead electrocardiogram, laboratory assessment including serum amino N-terminal pro brain natriuretic peptide (NT-proBNP), transthoracic echocardiography (TTE) and right heart catheterization (RHC) followed by coronary angiography^22^. Cardiac magnetic resonance (CMR) imaging was performed in a subgroup of patients (n = 157). All baseline examinations were performed in clinically stable patients without any clinical signs of decompensation^22^.

### Clinical endpoints

The primary endpoint was a combined endpoint defined as hospitalization due to HF and/or death from any cause. Secondary endpoints were hospitalization due to HF and all-cause mortality.

### Diagnostic definitions

According to the guidelines of the American College of Cardiology Foundation and American Heart Association^23^ and the European Society of Cardiology^24^, the following criteria had to be fulfilled to confirm the diagnosis of HFpEF: symptoms/signs of HF, LV ejection fraction (LVEF) ≥50%, diastolic dysfunction and/ or structural alterations (LV hypertrophy, left atrial enlargement) by TTE and serum NT-proBNP levels >220 pg/ml.

Patients with significant coronary artery disease (CAD, stenosis ≥50%) and significant valvular heart disease other than tricuspid regurgitation were excluded from the registry. Arterial hypertension was defined according to the recent guidelines with a systolic blood pressure ≥140 mmHg and/or diastolic blood pressure ≥90 mmHg^25^ or if the patient already received antihypertensive medication. Hyperlipidemia was defined as low-density lipoprotein cholesterol >100 mg/dl^26^ or if the patient was already on statins and/or ezetimibe prior to study inclusion.

Furthermore, patients with excessive alcohol intake were excluded and complete serologic assessment was performed in all patients to rule out any infectious, cholestatic or autoimmune hepatic disorder.

### Bioelectrical impedance spectroscopy

Bioelectrical impedance spectroscopy was performed using a portable whole-body device, the Body Composition Monitor (Fresenius Medical Care, Bad Homburg, Germany)^22^. Patients were placed in supine position and electrodes were attached to the nondominant hand and the ipsilateral foot. Measurements were conducted according to the manufacturer’s manual. The following parameters were obtained: fat tissue index (kg/m^2^), total fat mass (kg), relative fat mass (%, total body weight divided by total fat mass). Body composition monitoring was performed in 161 patients.

### Laboratory analysis

Complete blood count and blood chemistry including liver enzymes were performed as part of clinical routine assessment. Capillary blood from the earlobe was measured using an ABL 510 blood gas analyzer (Radiometer Medical ApS, Bronshoj, Denmark). Serum NT-proBNP was measured with an immunological test (Elecsys^®^ Systems, Roche Diagnostics, Rotkreuz, Switzerland). Upper limit of normal serum gamma-glutamyltransferase (GT) levels was 60 units per liter (U/L).

### Echocardiography

Board certified physicians performed TTE using high-end scanners such as GE Vivid 7 and E9 (GE Healthcare, Wauwatsa, WI, USA)^22^. All measurements were done according to the guidelines of the American Society of Echocardiography^27^. LVEF was assessed using the biplane Simpson’s method. Pulsed-wave Doppler was performed to obtain the early (E) to late (A) ventricular filling velocities. E´ (early diastolic mitral annular velocity) was assessed at the septal and lateral side of the mitral annulus with Tissue Doppler Imaging and averaged to calculate E/E’. Right ventricular (RV) function (RVF) was assessed by integrating visual assessment of contractility of the RV outflow tract, RV apex and interventricular septum from different views. Tricuspid regurgitation (TR) was quantified according to recent recommendations^28^. Moderate and severe TR were considered significant^29^.

### Cardiac catheterization

For hemodynamic confirmation of HFpEF, a 7F Swan-Ganz catheter (Baxter, Healthcare Corp, Munich, Germany) was inserted via a femoral approach^22^. CathCorLX (Siemens AG, Erlangen, Germany) was used to measure pressures, which were recorded as average of eight measurements over eight recorded heart cycles. Cardiac output (CO) was assessed by thermodilution and by Fick’ s method. The transpulmonary pressure gradient (TPG) was calculated by subtracting pulmonary artery wedge pressure (PAWP) from mean pulmonary artery pressure (PAP) and pulmonary vascular resistance (PVR) was calculated by dividing TPG by CO.

### Cardiac magnetic resonance

All CMR studies were performed by board-certified physicians on a 1.5-T cardiac-dedicated clinical magnetic resonance system (Avanto, Siemens Medical Solutions, Erlangen, Germany). The CMR protocol consisted of a functional study and late gadolinium enhancement (LGE) imaging and has been described previously by our group in more detail^30^. CMR was not performed in 117 patients due to claustrophobia, advanced stage renal disease, implanted pacemaker/implantable cardioverter defibrillator or other ferromagnetic implants.

### Statistical analysis

Statistical analysis was performed with IBM SPSS Statistics 23.0 (New York, USA). P-values from two-sided tests <0.05 were considered statistically significant. Data were expressed as mean ± standard deviation, median (interquartile range), or frequency and percent. Continuous variables were compared using the Student’s *t* –test or Wilcoxon rank-sum test, as appropriate. Differences between dichotomous variables were assessed using the χ^2^ test.

Univariable and multivariable Cox-regression models were calculated to examine factors associated with adverse outcome. Predictors in the multivariable Cox model were selected from the set of variables that reached statistical significance in univariable analysis. Logarithmic transformation was done in not-normally distributed variables prior to univariable calculations. Results were expressed as hazard ratios (HR) with 95% confidence intervals (CI).

Survival curves were estimated with the Kaplan-Meier method and log rank test was applied to compare survival differences.

The influence of relevant parameters on gamma-GT levels was investigated first by univariable logistic regression. To identify the most relevant predictors for each category (clinical, echocardiographic, hemodynamic, magnetic resonance imaging), a separate multiple regression model was selected from all variables that reached statistical significance in univariable analysis in the respective category by a stepwise procedure. Results were expressed as odds ratio (OR) with 95% CI.

## Results

### Clinical and cardiac characteristics at baseline

Between January 2011 and February 2017, 334 patients were referred. Of these, 18 patients were excluded because of relevant CAD, 20 because of cardiac amyloidosis and 15 had NT-proBNP levels below the inclusion threshold of 220 pg/ml. Additionally, 7 patients were excluded because of excessive alcohol intake and the suspicion of hepatitis.

Finally, 274 patients with confirmed HFpEF were enrolled. Mean age was 71.3 ± 8.4 years and 69.3% were female (Table 1). After 21.5 ± 18.6 months of follow-up, 97 patients (35.4%) reached the combined endpoint. These patients presented with higher New York Heart Association (NYHA) functional class (p < 0.001) at baseline, had more prior HF hospitalizations (p < 0.001), more frequently suffered from atrial fibrillation (p = 0.022), diabetes (p = 0.028) and chronic obstructive pulmonary disease (p = 0.016) and had a higher intake of diuretics (p = 0.006) compared with the remainder of the group (n = 177).

**Table 1:** Baseline characteristics.

	Total (n = 274)	Event (n = 97)	No Event (n = 177)	p-value
**Clinical parameters**				
Age, years	71.3 ± 8.4	71.9 ± 8.3	70.9 ± 8.5	0.330
Female, n (%)	190 (69.3)	62 (63.9)	128 (72.3)	0.171
Body Mass Index, kg/m^2^	30.5 ± 6.8	31.2 ± 7.7	30.1 ± 6.2	0.196
Fat Tissue Index, kg/ m^2^	15.9 ± 6.4	16.1 ± 7.0	15.9 ± 6.1	0.877
Total Fat Mass, kg	32.7 ± 12.9	34.1 ± 14.4	31.8 ± 11.8	0.272
Relative Fat Mass, %	37.9 ± 9.7	37.2 ± 10.1	38.4 ± 9.4	0.418
NYHA class III and IV, n (%)	181 (66.1)	80 (82.5)	101 (57.1)	<**0**.**001**
Prior HF hospitalization, n (%)	81 (29.6)	56 (57.3)	25 (14.1)	<**0**.**001**
Atrial fibrillation, n (%)	158 (57.7)	65 (67.0)	93 (52.5)	**0**.**022**
Arterial hypertension, n (%)	263 (96.0)	93 (95.9)	170 (96.0)	1.000
Hyperlipidemia, n (%)	153 (55.8)	51 (52.6)	102 (57.6)	0.447
Diabetes mellitus, n (%)	103 (37.6)	45 (46.4)	58 (32.8)	**0**.**028**
History of CAD, n (%)	67 (24.5)	24 (24.7)	43 (24.3)	1.000
COPD, n (%)	92 (33.6)	42 (43.3)	50 (28.2)	**0**.**016**
ACE-inhibitor, n (%)	82 (29.9)	34 (35.1)	48 (27.1)	0.214
ATII-blocker, n (%)	104 (37.9)	33 (34.0)	71 (40.1)	0.363
Calcium-antagonist, n (%)	81 (29.6)	28 (28.9)	53 (29.9)	0.702
Beta-blocker, n (%)	206 (75.2)	77 (79.4)	129 (72.9)	0.246
Oral anticoagulation, n (%)	218 (79.6)	81 (83.5)	137 (77.4)	0.274
Diuretic, n (%)	214 (78.1)	85 (87.6)	129 (72.9)	**0**.**006**
Statin, n (%)	134 (48.9)	44 (45.4)	90 (50.8)	0.449
**Echocardiographic parameters**				
LA diameter, mm	62.4 ± 7.9	63.1 ± 7.7	61.9 ± 8.1	0.254
LA indexed for BSA, ml/m^2^	51.3 ± 19.9	53.2 ± 17.2	50.5 ± 21.1	0.579
LVEDD, mm	44.0 ± 5.5	44.1 ± 6.0	43.9 ± 5.1	0.834
RA diameter, mm	62.6 ± 8.9	63.6 ± 9.2	61.9 ± 8.6	0.148
RVEDD, mm	37.0 ± 7.6	39.0 ± 8.5	35.7 ± 6.6	**0**.**001**
IVS, mm	12.9 ± 2.6	12.9 ± 2.3	12.9 ± 2.9	0.797
E/E’ ratio	15.3 ± 6.3	16.4 ± 8.0	14.8 ± 5.4	0.318
E/A ratio	1.6 ± 1.1	1.8 ± 0.9	1.6 ± 1.1	0.469
Significant TR, n (%)	136 (49.6)	60 (61.9)	76 (42.9)	0.108
**Hemodynamic parameters**				
Systolic PAP, mmHg	53.9 ± 17.5	61.4 ± 17.0	49.6 ± 16.4	**<0**.**001**
Diastolic PAP, mmHg	22.3 ± 7.5	24.8 ± 6.7	20.9 ± 7.7	**<0**.**001**
Mean PAP, mmHg	34.4 ± 10.2	38.4 ± 9.1	32.1 ± 10.0	**<0**.**001**
Mean RAP, mmHg	12.6 ± 5.6	14.3 ± 6.2	11.6 ± 5.0	**0**.**001**
PAWP, mmHg	20.1 ± 5.9	21.8 ± 5.8	19.1 ± 5.7	**0**.**001**
SaO_2_, %	93.8 ± 4.7	92.8 ± 4.9	94.3 ± 4.5	**0**.**018**
TPG, mmHg	14.3 ± 7.5	16.6 ± 8.3	12.9 ± 6.6	**<0**.**001**
PVR, dynes.s.cm^−5^	228.1 ± 136.4	264.3 ± 151.3	206.8 ± 122.5	**0**.**002**
SV, ml	73.5 ± 21.4	75.7 ± 22.8	72.3 ± 20.6	0.260
CO thermodilution, l/min	5.3 ± 1.4	5.3 ± 1.4	5.2 ± 1.4	0.748
CO Fick, l/min	4.5 ± 1.3	4.4 ± 1.2	4.6 ± 1.3	0.401
**Magnetic resonance imaging parameters** **(n** = **157) (n** = **59) (n** = **98)**				
LA, mm	65.6 ± 9.4	67.8 ± 9.4	64.2 ± 9.2	**0**.**021**
LVEDV, ml	126.9 ± 44.8	131.2 ± 45.6	124.4 ± 44.4	0.371
RA, mm	65.6 ± 9.2	66.9 ± 10.2	64.8 ± 8.4	0.157
RVEDV, ml	160.7 ± 102.2	171.9 ± 72.5	153.9 ± 116.1	0.294
IVS, mm	11.2 ± 2.1	11.3 ± 1.7	11.1 ± 2.3	0.506
LVEF, %	53.3 ± 13.8	53.7 ± 14.3	53.0 ± 13.5	0.715
RVEF, %	51.8 ± 11.3	48.1 ± 11.6	53.9 ± 10.7	**0**.**002**

Continuous values are shown as mean ± standard deviation; A - mitral peak velocity of late filling, ACE – angiotensin converting enzyme, AT – angiotensin, BSA – body surface area, CAD - coronary artery disease, CO - cardiac output, COPD - chronic obstructive pulmonary disease, E - early mitral inflow velocity, E’ - early diastolic mitral annular velocity, ECV - extra cellular volume, HF – heart failure, IVS - interventricular septum, LA - left atrial, LVEDV - left ventricular end-diastolic volume, LVEF - left ventricular ejection fraction, NYHA - New York Heart Association, PAP - pulmonary artery pressure, PAWP - pulmonary artery wedge pressure, PVR - pulmonary vascular resistance, RA - right atrial, RAP - right atrial pressure, RVEDV - right ventricular end-diastolic volume, RVEF - right ventricular ejection fraction, SaO_2_ - arterial saturation of oxygen, SV - stroke volume, TPG - transpulmonary gradient, TR – tricuspid regurgitation.

RV end-diastolic diameter (RVEDD) as assessed by TTE was significantly larger in the event cohort (p = 0.001), which also displayed higher pulmonary artery pressures (e.g. mean PAP, p < 0.001), lower oxygen saturation (p = 0.018), higher TPG (p < 0.001) and higher PVR (p = 0.002).

In a subgroup of 157 patients who underwent CMR, left atrial (LA) size was larger in the event group (p = 0.021). Furthermore, on average, RV ejection fraction (RVEF) was significantly lower in this cohort (p = 0.002).

### Liver enzymes, other laboratory parameters and outcome

Patients in the event group presented with lower hemoglobin levels (p = 0.001), lower serum iron levels (p = 0.001) and higher levels of C-reactive protein (p = 0.013). Furthermore, glomerular filtration rates were lower (p < 0.001) and NT-proBNP levels (p = 0.001) as well as serum gamma-GT levels (p < 0.001) were higher in patients who had reached the combined endpoint. No difference could be found with respect to other cholestatic parameters (p = 0.126 for alkaline phosphatase, p = 0.774 for bilirubin). Interestingly, patients in the event group showed lower levels of total cholesterol (p = 0.030).

Multivariable Cox-regression analysis was calculated using eight laboratory parameters that were statistically significant in univariable analysis. In the multivariable model, hemoglobin (p = 0.006), serum gamma-GT (p = 0.004) and serum NT-proBNP (p = 0.001) were associated with worse clinical outcome (Table 2).

**Table 2:** Laboratory parameters and their association with clinical outcome.

	Total (n = 274)	Event (n = 97)	No Event (n = 177)	Univariable		Multivariable	
				HR (95% CI)	p-value	HR (95% CI)	p-value
Hemoglobin, mg/dl	12.5 ± 1.7	12.0 ± 1.8	12.7 ± 1.6	0.796 (0.704–0.899)	<0.001	0.840 (0.741–0.952)	**0**.**006**
Platelet count, G/L	233.9 ± 75.1	244.9 ± 84.8	227.8 ± 68.7	1.002 (1.000–1.005)	0.053		
White blood count, G/L	7.1 (5.7–8.7)	7.1 (5.7–9.6)	7.1 (5.8–8.3)	2.044 (0.493–8.472)	0.324		
Serum iron, μg/dl	66.0 (46.5–95.5)	57.0 (42.0–79.0)	74.5 (49.0–104.3)	0.364 (0.196–0.675)	0.001		
Total bilirubin, mg/dl	0.6 (0.4–0.9)	0.6 (0.5–0.9)	0.5 (0.4–0.9)	1.235 (0.559–2.725)	0.602		
Albumin, mg/dl	40.6 ± 4.9	40.1 ± 5.6	41.0 ± 4.4	0.964 (0.922–1.008)	0.105		
Lipase, U/L	31.0 (22.0–45.0)	32.0 (21.0–43.0)	31.0 (22.0–46.0)	1.136 (0.517–2.498)	0.751		
Alkaline phosphatase, U/L	77.0 (62.0–98.0)	81.0 (64.5–113.5)	75.0 (61.0–92.5)	1.770 (0.544–5.759)	0.343		
ASAT, U/L	25.0 (20.0–31.3)	24.0 (19.5–31.0)	25.0 (21.0–32.0)	0.413 (0.105–1.624)	0.205		
ALAT, U/L	22.0 (16.0–29.0)	19.0 (14.0–26.0)	23.0 (18.0–31.0)	0.175 (0.064–0.482)	0.001		
Gamma-GT, U/L	36.0 (22.0–66.0)	49.0 (26.5–100.5)	33.0 (21.0–51.0)	2.499 (1.544–4.044)	< 0.001	1.002 (1.001–1.003)	**0**.**004**
LDH, U/L	215.0 (185.0–253.0)	222.0 (185.0–273.5)	211.0 (185.0–246.5)	3.546 (0.536–23.473)	0.189		
C-reactive protein, mg/dl	0.4 (0.2–0.9)	0.6 (0.2–1.4)	0.4 (0.2–0.8)	1.814 (1.236–2.663)	0.002		
Cholesterol, mg/dl	171.1 ± 41.4	163.8 ± 43.3	175.2 ± 39.8	0.992 (0.988–0.997)	0.001		
Triglycerides, mg/dl	113.0 (83.0–155.0)	113.0 (89.0–158.8)	116.0 (81.0–153.0)	0.758 (0.269–2.139)	0.601		
GFR, ml/min/1.73 m^2^	59.4 ± 19.9	52.4 ± 17.0	63.2 ± 20.4	0.977 (0.967–0.987)	<0.001		
HbA1c, %	5.9 (5.6–6.5)	6.1 (5.7–6.7)	5.9 (5.6–6.5)	2.326 (0.573–9.442)	0.238		
NT-proBNP quartile (pg/ml)				3.015 (1.926–4.719)	<0.001	2.213 (1.373–3.569)	**0**.**001**
0–600, n (%)	89 (32.5)	20 (20.6)	69 (38.9)				
601–1200, n (%)	59 (21.5)	11 (11.3)	48 (27.1)				
1201–1800, n (%)	48 (17.5)	22 (22.7)	26 (14.7)				
>1800, n (%)	78 (28.5)	44 (45.4)	34 (19.3)				

Continuous values are shown as mean ± standard deviation or median (interquartile range); ALAT - Alanin Aminotransferase, ASAT - Aspartat Aminotransferase, GFR - Glomerular Filtration Rate, GT - Glutamyl Transferase, LDH - Lactatdehydrogenase, NT-proBNP - N-terminal pro Brain Natriuretic Peptide.

**Figure 1:**
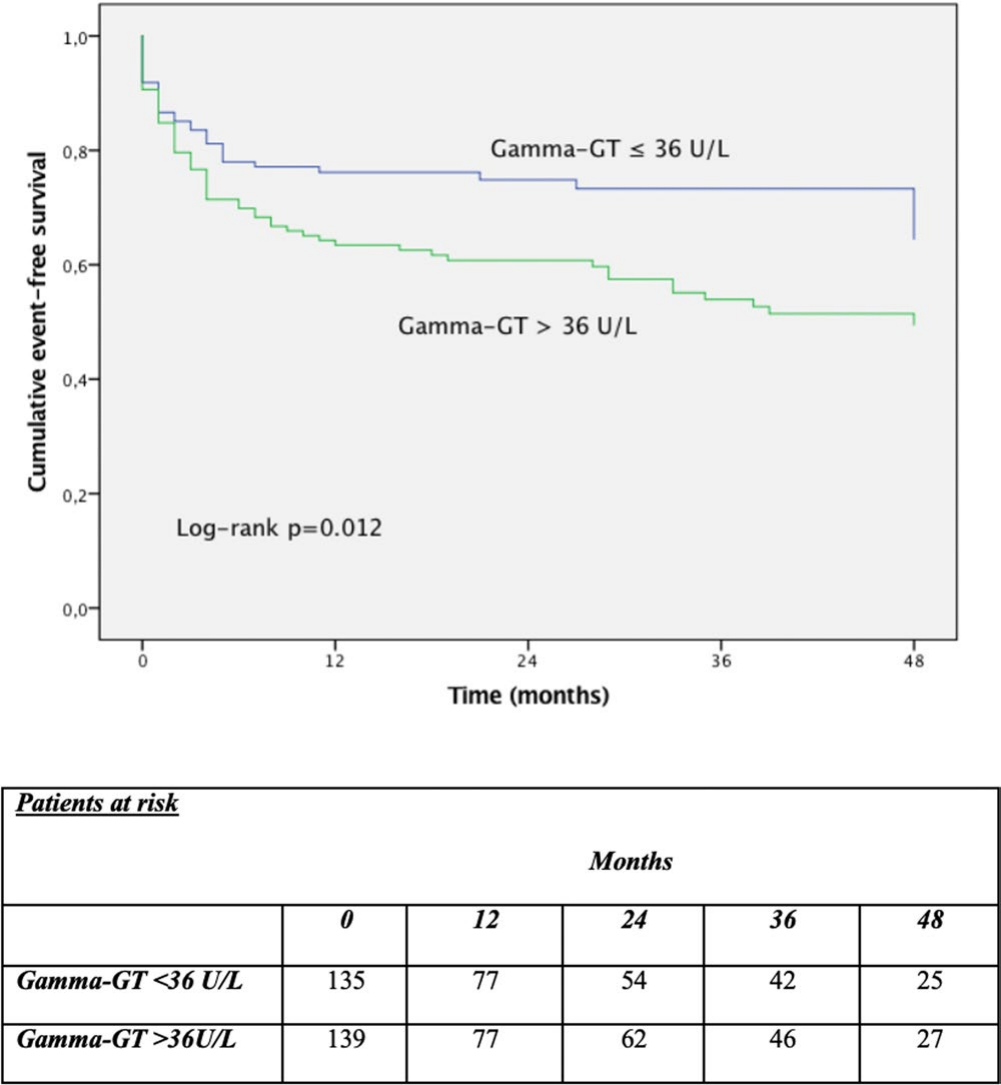
Kaplan-Meier event-free survival curve according to the gamma glutamyltransferase (GT) median of 36 U/L.

**Figure 2:**
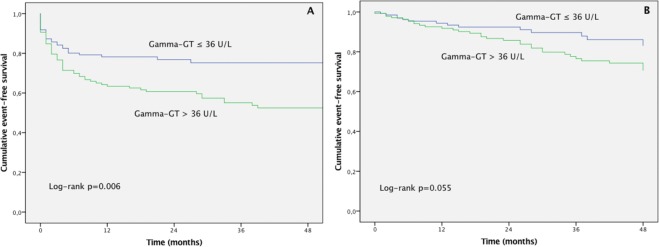
Kaplan-Meier curves of hospitalization due to heart failure worsening (**A**) and all-cause mortality (**B**).

Kaplan-Meier analysis of the combined primary endpoint showed that patients with gamma-GT levels >36 U/L had significantly more events compared to patients with levels ≤ 36 U/L (log-rank p = 0.012, Fig. 1). Secondary endpoint analysis showed a significant difference in HF hospitalizations (log-rank p = 0.006) and a trend in all-cause mortality (log-rank p = 0.055, Fig. 2).

### Determinants of gamma-glutamyltransferase

Univariable logistic regression was performed for each category to identify parameters associated with gamma-GT. The following variables were statistically significant in the univariable model: *clinical*: atrial fibrillation (OR 3.140, p < 0.001); *echocardiographic*: LA diameter (OR 1.061, p = 0.001), RA diameter (OR 1.064, p < 0.001), RVEDD (OR 1.060, p = 0.002), E/A ratio (OR 2.173, p = 0.024) and significant TR (OR 3.153, p < 0.001); *hemodynamic*: systolic PAP (OR 1.027, p = 0.001), diastolic PAP (OR 1.069, p = 0.001), mean PAP (OR 1.062, p < 0.001), mean RA pressure (OR 1.140, p < 0.001), PAWP (OR 1.110, p < 0.001), TPG (OR 1.044, p = 0.025), PVR (OR 1.002, p = 0.048); *magnetic resonance imaging*: LA diameter (OR 1.036, p = 0.050), RA diameter (OR 1.052, p = 0.009), RV end-diastolic volume (OR 1.012, p = 0.001) and RVEF (OR 0.963, p = 0.014).

By multivariable logistic regression analysis of each category, *echocardiographic* (*5 variables)*: E/A ratio (OR 2.173, p = 0.024), *hemodynamic* (*7 variables)*: mean RA pressure (OR 1.139, p < 0.001) and *magnetic resonance imaging* (*4 variables)*: RA diameter (OR 1.070, p = 0.001) remained independently associated with gamma-GT levels (Table 3).

**Table 3:** Parameters associated with gamma-glutamyltransferase (GT).

	Univariable		Multivariable	
	Odds Ratio (95% CI)	p-value	Odds Ratio (95% CI)	p-value
***Clinical parameters***				
Age	0.988 (0.960–1.016)	0.397		
Female	1.669 (0.992–2.809)	0.054		
Body Mass Index	1.021 (0.986–1.059)	0.243		
Fat Tissue Index	0.989 (0.942–1.038)	0.641		
Total Fat Mass	0.990 (0.967–1.015)	0.438		
Relative Fat Mass	0.975 (0.943–1.008)	0.133		
Systolic blood pressure	0.994 (0.982–1.006)	0.308		
Diastolic blood pressure	1.004 (0.985–1.024)	0.661		
Prior HF hospitalization	1.702 (0.950–3.049)	0.074		
Atrial fibrillation	3.140 (1.902–5.181)	<0.001		
Hypertension	1.846 (0.528–6.4559	0.337		
Hyperlipidemia	0.807 (0.500–1.301)	0.379		
Diabetes mellitus	1.263 (0.774–2.062)	0.350		
History of CAD	0.729 (0.419–1.268)	0.263		
COPD	1.420 (0.857–2.351)	0.173		
***Echocardiographic parameters***				
LA diameter	1.061 (1.024–1.099)	0.001		
LA indexed for BSA	1.023 (0.999–1.047)	0.059		
LVEDD	0.994 (0.948–1.042)	0.801		
RA diameter	1.064 (1.030–1.100)	<0.001		
RVEDD	1.060 (1.021–1.101)	0.002		
IVS	1.009 (0.915–1.112)	0.863		
E/E’ ratio	1.033 (0.959–1.112)	0.395		
E/A ratio	2.173 (1.108–4.260)	0.024	2.173 (1.108–4.260)	**0**.**024**
Significant TR	3.153 (1.846–5.385)	<0.001		
***Hemodynamic parameters***				
Systolic PAP	1.027 (1.011–1.044)	0.001		
Diastolic PAP	1.069 (1.029–1.112)	0.001		
Mean PAP	1.062 (1.031–1.094)	<0.001		
Mean RAP	1.140 (1.078–1.206)	<0.001	1.139 (1.076–1.205)	<**0**.**001**
PAWP	1.110 (1.055–1.168)	<0.001		
SaO2	1.041 (0.982–1.102)	0.176		
TPG	1.044 (1.005–1.084)	0.025		
PVR	1.002 (1.000–1.004)	0.048		
SV	0.996 (0.983–1.008)	0.496		
CO thermodilution	1.037 (0.856–1.258)	0.709		
CO Fick	0.898 (0.714–1.130)	0.358		
***Magnetic resonance imaging parameters***				
LA	1.036 (1.000–1.073)	0.050		
LVEDV	1.006 (0.998–1.014)	0.132		
RA	1.052 (1.013–1.092)	0.009	1.070 (1.026–1.115)	**0**.**001**
RVEDV	1.012 (1.005–1.019)	0.001		
IVS	1.022 (0.879–1.188)	0.779		
LVEF	1.000 (0.983–1.017)	0.982		
RVEF	0.963 (0.935–0.992)	0.014		

A - mitral peak velocity of late filling, CAD - coronary artery disease, CO - cardiac output, COPD - chronic obstructive pulmonary disease, E - early mitral inflow velocity, E’ - early diastolic mitral annular velocity, ECV - extra cellular volume, HF – heart failure, IVS - inter-ventricular septum, LA - left atrial, LVEDV - left ventricular end-diastolic volume, LVEF - left ventricular ejection fraction, PAP - pulmonary artery pressure, PAWP - pulmonary artery wedge pressure, PVR - pulmonary vascular resistance, RA - right atrial, RAP - right atrial pressure, RVEDV - right ventricular end-diastolic volume, RVEF - right ventricular ejection fraction, SaO_2_ - arterial saturation of oxygen, SV - stroke volume, TPG - transpulmonary gradient, TR – tricuspid regurgitation.

## Discussion

Our analysis of a well-characterized HFpEF patient cohort is the first to identify serum gamma-GT levels as an independent predictor of clinical outcome, besides hemoglobin and NT-proBNP.

Although we found a clear association between gamma-GT serum levels and the degree of LV diastolic function as well as blood pressures in the RA, the exact underlying pathophysiologic mechanisms remain yet unclear. It is not entirely possible to discern whether liver congestion as a result of elevated left and right-sided cardiac filling pressures or systemic low-grade inflammation with consecutive cardiac stiffness and pressure rise, or both mechanisms drive gamma-GT alteration in HFpEF patients.

### Evidence supporting hepatic congestion as a determinant of serum gamma-glutamyltransferase levels

In 2000, Naschitz *et al*. systematically performed a survey of the Medline database and were the first to describe the close relationship between cardiac and hepatic disorders^8^. Over the past decades, there has been growing evidence on the importance of liver enzymes and abnormal liver function tests (LFT) in HF, especially in patients with reduced EF. Van Deursen and co-workers analyzed 234 ADHF patients and showed that abnormal LFTs were associated with an increased risk for mortality, rehospitalization, and in-hospital worsening of HF^13^. This finding was recently confirmed in the PROTECT study, where abnormal levels of Aspartat Aminotransferase (ASAT), Alanin Aminotransferase (ALAT) and bilirubin were associated with a higher-risk of in-hospital mortality and 180-day mortality^11^. In the larger ASCEND-HF trial, LFT data were obtained from 4.228 patients and more than 40% of study participants were out of range at the time of hospital admission, but only bilirubin was independently associated with worse clinical outcome^12^, which has also been confirmed in the CHARM program a few years before^18^ identifying bilirubin as a sensitive marker for congestion in HF with reduced EF. The crucial prognostic role of total bilirubin has also been described in the EVEREST trial in 2012^10^, but Okada and the NaDEF investigators performed a more detailed assessment of bilirubin fractionation and found direct bilirubin superior to total bilirubin in predicting outcome in ADHF^16^.

Nevertheless, there is limited evidence on liver enzymes and LFTs in HF patients with preserved ejection fraction. In our cohort of 274 patients, we identified gamma-GT as an independent predictor of outcome after a mean follow-up of approximately two years and did not find any association with bilirubin or other LFTs. Interestingly, in previous observations gamma-GT was associated with in-hospital mortality in 183 ADHF patients with an ejection fraction < 50%^31^, but no association with long-term outcome has yet been reported, especially not in HFpEF patients. In 2002, Lau *et al*. showed that gamma-GT and alkaline phosphatase increased in direct proportion to the severity of TR in HF patients^32^. Significant TR is a common finding in HFpEF and was present in 49.6% of our patients and there was an association between TR and gamma-GT levels, if only in univariable analysis.

We were able to show that elevated RA filling pressures and higher RA diameters, as assessed by CMR, were associated with changes in gamma-GT. This is in accordance with a recently published study of Taniguchi and co-workers, who suggested that liver stiffness, reflecting right-sided filling pressures, provides important information regarding patients’ volume status^33^. Furthermore, the authors of this study concluded that liver congestion at hospital discharge is linked to HF hospitalization and death, although, once more, performed in patients with reduced systolic function^33^.

### Evidence supporting low-grade inflammation as a determinant of serum gamma-glutamyltransferase levels

In 2011, Kanbay *et al*. evaluated the medical records of 166 patients with obstructive sleep apnea syndrome and found high serum gamma-GT levels as an independent predictor of cardiovascular disease^34^, supporting the theory of gamma-GT as a ‘novel’ cardiovascular biomarker^35^ and as a factor associated with subclinical inflammation^36^. In line with this, patients who have reached the combined endpoint in our analysis had slightly higher levels of C-reactive protein. These findings are in accordance with the hypothesis of Paulus and Tschöpe that chronic low-grade inflammation processes due to a variety of comorbidities lead to alterations in myocardial structure and function resulting in abnormal LV relaxation and consecutive diastolic dysfunction^37^. This theory was confirmed by Mantovani *et al*., who were able to show that in patients with type-2 diabetes, the inflammatory state of NAFLD was associated with early LV diastolic dysfunction^21^, which has been identified as an independent predictor of reduced functional capacity^22^.

Additionally, patients with NAFLD were more likely to be female and overweight/obese^21^ which is in line with a previous finding from our group supporting the HFpEF obesity phenotype^22,38,39^. A recently published analysis suggested that an increase in gamma-GT concentrations is a sensitive and early biomarker of unfavorable body fat distribution even in healthy individuals^40^. The median serum level in this study was 21.6 U/L compared to 36.0 U/L in our cohort, which is quite in the range of normal values. Thus, even gamma-GT levels within the normal range may provide important information concerning the prognosis of HF patients with preserved EF , although these results need to be confirmed in larger registries and clinical trials.

## Limitations

Due to the single-center design, a center-specific bias cannot be excluded. In addition, liver sonographies and measurement of liver stiffness by elastography^41^ have not been systematically performed. Therefore, structural hepatic alterations and the specific impact of fatty liver disease^42^ cannot be accurately determined. However, we found a clear association between HFpEF-defining cardiac parameters and gamma-GT serum levels, while no such relation could be found with other liver function parameters. More information on the relation between gamma-GT and cardiac function as well as its association with other biomarkers reflecting pathophysiological processes is needed to further investigate its clinical utility.

Finally, due to the relatively small patient population, our results should be interpreted as hypothesis-generating and certainly need confirmation in larger patient collectives.

## Conclusion

Our study demonstrates that serum levels of gamma-GT in HFpEF patients are multifactorial and largely related to LV diastolic dysfunction as well as right-sided heart alterations.

Serum gamma-GT levels may serve as an easily available tool to predict clinical prognosis, especially in the absence of further investigative modalities. Importantly, next to NT-proBNP and hemoglobin, serum gamma-GT levels predicted hospitalization and mortality in HFpEF patients.

## Data Availability

All data generated or analysed during this study are included in this published article (and its Supplementary Information files).
